# The Future of Pediatric Dentistry Is Now

**DOI:** 10.3390/children10010097

**Published:** 2023-01-03

**Authors:** Maria Grazia Cagetti, Guglielmo Campus

**Affiliations:** 1 Department of Biomedical, Surgical and Dental Sciences, University of Milan, 20122 Milan, Italy; 2 Department of Restorative, Preventive and Paediatric Dentistry, School of Dental Medicine, University of Bern, 3010 Bern, Switzerland

For decades, pediatric dentistry was considered the Cinderella of all dental disciplines. Not because its role in maintaining and/or restoring children’s oral health was not recognized, but because compared to other disciplines where new technologies and the resulting economic impact brought constant changes and improvements in dental procedures, it seemed that pediatric dentistry was not evolving [1]. This is not true, especially in today’s dental landscape.

It is undeniable that an individual’s oral health is built during childhood [2]. Correct eating, hygiene and behavioral habits lead, in most cases, to the development of a healthy and harmonious dental, periodontal and skeletal apparatus. Moreover, thanks to the mass introduction of fluoride, caries has decreased worldwide [3]. Nevertheless, it remains, even today, a highly frequent disease in childhood. Several carious lesions, especially in deciduous dentition, affect disadvantaged children and often go untreated [4]. From this perspective, the paradigm shift that cariologists are suggesting and that dentists are still struggling to accept, i.e., considering caries as a disease that should not be treated with a drill and instead modifying the risk factors, is bringing and will bring great benefits to children’s health [5]. The widespread use of non-operative and minimally invasive treatments for lesion management is changing the clinical routine of physicians and their patients.

The pediatric dentist, unlike the dentist who primarily treats adults, must be familiar with and apply behavioral techniques and, if necessary, pharmacological in order to provide treatment reducing anxiety thanks to “tailored” approaches for each patient [6,7]. Visual pedagogy, exploiting the new virtual reality technologies and even hypnosis, allow the pediatric dentist to reduce the stress of the young patient and, consequently, of the family and the dental team [8,9]. These behavioral strategies make it possible to reduce the use of general anesthesia, which has been the gold standard for providing dental care to a very young patient or those with special needs. In addition, pediatric dentists need to be able to face daily challenges, such as the increasing impact on the oral health of the development defects of enamel or the treatment of complex situations in the context of rare syndromes [10,11].

Turning to the more innovative aspects of the profession, the introduction of new preventive and restorative materials that are better suited to the needs of young patients represents an important milestone [12]. The introduction of moisture-tolerant resin-based materials makes it possible to work with a higher probability of success even when a perfectly moisture-free field is not possible [13]. Working under a rubber dam is mandatory for all procedures requiring chemical adhesion, but those who work ‘in the trenches’ with children know that this is not always possible.

Oral health includes the ability to speak, smile, smell, taste, touch, chew, swallow and convey a range of emotions through facial expressions with confidence, without pain or discomfort [14]. If one or more of these functions are impaired, an individual’s daily well-being and overall quality of life are compromised [15]. Dental appearance may significantly affect children and their families’ social and emotional well-being [16]. Dentistry cannot, therefore, shy away from offering functionally but also aesthetically effective solutions to children [17,18].

It is difficult to say what further changes we can expect. What we must bear in mind, however, is that the child must be placed at the center of care, must be listened to in his or her needs, understood in his or her difficulties and limitations and helped to become a healthy adult. With these purposes in mind, we organized a weekly Summer School in Sardinia (Italy), where teachers and participants discuss these issues with mutual enrichment. (https://www.sardiniameeting.it/en/event/xv-summer-school-of-the-ccwho-of-milan, accessed on 20 December 2022).

This collection deals with different and distant aspects of pediatric dentistry, but with a common denominator: the oral and general well-being of children [19]. The papers in this collection hope to inspire researchers to think more broadly when addressing children’s oral health.
